# Parkinson’s Disease-Associated Mutant LRRK2-Mediated Inhibition of miRNA Activity is Antagonized by TRIM32

**DOI:** 10.1007/s12035-017-0570-y

**Published:** 2017-05-15

**Authors:** Laura Gonzalez-Cano, Ingeborg Menzl, Johan Tisserand, Sarah Nicklas, Jens C. Schwamborn

**Affiliations:** Luxembourg Centre for Systems Biomedicine (LCSB), University of Luxembourg, 6, Avenue du Swing, 4367 Belvaux, Luxembourg

**Keywords:** Parkinson’s disease, TRIM32, LRRK2, miRNA activity, Neuronal differentiation

## Abstract

Parkinson’s disease (PD) is the second most common neurodegenerative disorder. Accumulating evidences suggest that PD might have a strong neurodevelopmental component. Among the genetic cases, mutations in the leucine-rich repeat kinase 2 (LRRK2) are well known to be disease causing. Although the molecular mechanism of the pathogenic LRRK2 function is not fully clear, inhibition of microRNA (miRNA) activity has been suggested to be among the pathogenic LRRK2 targets. Here, we demonstrate that the miRNA activity inhibition function of pathogenic LRRK2 is directly antagonized by the neuronal cell fate determinant TRIM32. These findings suggest that TRIM32 might be a modifier for PD and could be a novel therapeutic target.

**Electronic supplementary material:**

The online version of this article (doi:10.1007/s12035-017-0570-y) contains supplementary material, which is available to authorized users.

## Introduction

Parkinson’s disease (PD) is the second most common neurodegenerative disorder [1]. The main pathologic hallmark of PD is the degeneration of dopaminergic neurons in the substantia nigra of the midbrain, but it is now accepted that the disease is way more complex and that multiple brain regions and organs beyond the brain are affected [2–4]. Accumulating evidences even suggest that PD might have a strong neurodevelopmental component, implying that deregulated processes during embryonic development lead to PD, which determines the anlagen or susceptibility to develop the disease [5–7]. This anlage might be compensated for a long time before symptoms develop at higher ages.

Currently, about 15% of PD patients have a monogenic disease caused by one of the known mutations in the 15 associated genes; additionally, 25 genetic risk factors have been identified [8]. Leucine-rich repeat kinase 2 (LRRK2) is probably the best studied PD-associated gene [9]. Interestingly, PD-associated mutations in this gene only cause the disease in about 30% of the carriers [10]. The latter suggests that other genetic or environmental factors strongly contribute to the development of PD. Recently, it has been demonstrated in *Drosophila* that pathogenic LRRK2 inhibits microRNA (miRNA)-mediated translational repression [11]. Interestingly, important cell cycle regulators were affected by this altered translational control. This observation further supports the concept that PD-associated genes play a significant function in developmentally important processes like cell cycle control. It is tempting to speculate that pathogenic LRRK2, via miRNA activity modification, affects the cell cycle in neural stem cells and thereby alters the fate specification of neurons during development. This effect of pathogenic LRRK2 might result only in a slight delay in the neuronal differentiation process. However, eventually this delay is probably enough to alter the complex neuronal system, which might be the basis for increasing neurodegeneration susceptibility at a later stage.

In contrast to LRRK2, the neuronal cell fate determinant TRIM32 has been described as an activator of miRNA-mediated translational repression [12, 13]. TRIM32 belongs to the TRIM-NHL family of proteins that is characterized by the presence of an N-terminal RING finger, one or two B boxes, a coiled-coil region, and a C-terminal NHL domain [14]. This conserved protein family has been implicated in diverse biological processes, such as developmental timing, cell cycle progression, transcriptional regulation, and apoptosis [15]. Previously, we have shown that TRIM32 suppresses proliferation and induces neuronal differentiation in NSCs from embryonic [13, 16, 17] and adult mouse brain [18]. Through its C-terminal NHL domain, TRIM32 directly binds to miRNA-associated proteins of the Argonaute family, which leads to enhanced activity of specific microRNAs including Let-7a [12, 13]. Interestingly, TRIM32 has been implicated as regulator or target of the PD-associated genes alpha-synuclein and parkin [19, 20].

Here, we demonstrate that mammalian LRRK2 directly interacts with the Argonaute-2 protein and that pathogenic mutant LRRK2 inhibits the activity of the miRNA Let-7a. We further show that the effect of pathogenic LRRK2 is directly antagonized by TRIM32. These results suggest TRIM32 as a novel target for Parkinson’s disease-modifying therapies.

## Results and Discussion

### LRRK2, TRIM32, and Ago2 Interact In Vitro and In Vivo

**Fig. 1:**
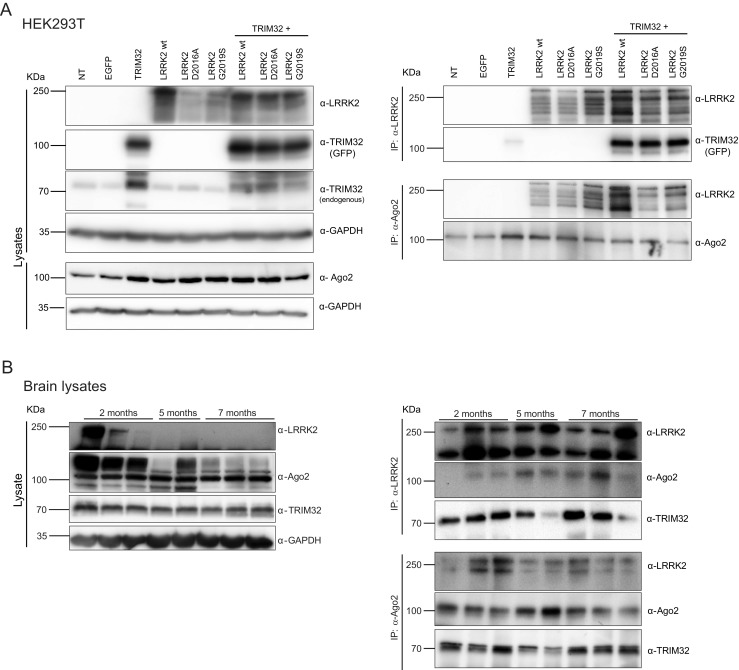
TRIM32, LRRK2, and Ago2 form a complex. **a** HEK293T cells were transfected with plasmids for the overexpression of the indicated constructs. On the *left panel*, immunoblots of cell lysates, probed with the indicated antibodies, are shown. On the *right panel*, immunoprecipitations with anti-LRRK2 and anti-Ago2 antibodies are shown. The blots are probed with the indicated antibodies. **b** On *the left panel*, immunoblots of lysates from brains of mice at different ages displaying the expression levels of endogenous LRRK2, Ago2, and TRIM32. On the *right panel*, immunoprecipitations with anti-LRRK2 and anti-Ago2 antibodies are shown. The blots are probed with the indicated antibodies

Previously, it has been shown in *Drosophila* that LRRK2 interacts with Argonaute proteins [11]. In the first set of experiments, we wanted to confirm if this interaction is conserved in mammals. If so, we aimed at addressing whether it depends on the presence of the PD-associated G2019S mutation in LRRK2, which increases the kinase activity [21], or the kinase dead mutation, D2016A. Furthermore, we were interested in determining the presence of TRIM32 in the LRRK2/Ago2 complex. Therefore, we co-transfected HEK293T cells, which endogenously express Ago2, with plasmids encoding for GFP-TRIM32 together with different Flag-LRRK2 constructs (Fig. 1a, Supplementary Fig. 1A). Via immunoprecipitation (IP) assays, we saw that LRRK2 is able to co-precipitate TRIM32 and that Ago2 co-precipitates with LRRK2 (Fig. 1a, Supplementary Fig. 1A). None of these interactions was significantly affected by the presence of LRRK2 mutations. Furthermore, the co-expression of TRIM32 also had no detectable effect on the stability of LRRK2 or Ago2 (Supplementary Fig. 1b).

After demonstrating the existence of a LRRK2/Ago2/TRIM32 complex in vitro, we wanted to verify this interaction with endogenously present proteins in vivo. Therefore, we used brain lysates from mice of 2, 5, and 7 months of age. Interestingly, we observed a strong downregulation of LRRK2 during this time period (Fig. 1b). A similar age-associated downregulation of LRRK2 has been observed before in the mouse spleen [22]. This observation further supports the hypothesis that LRRK2 has an important role during development. Again, via IP assays with antibodies directed against either LRRK2 or Ago2, we were able to show an interaction between LRRK2, Ago2, and TRIM32 (Fig. 1b). None of these interactions was significantly affected by the age of the animals. With this first set of experiments, we have provided evidences that LRRK2, Ago2, and TRIM32 form a complex in vitro and in vivo.

### TRIM32 and Mutant LRRK2 Bind Independently at the RNA-Induced Silencing Complex

**Fig. 2:**
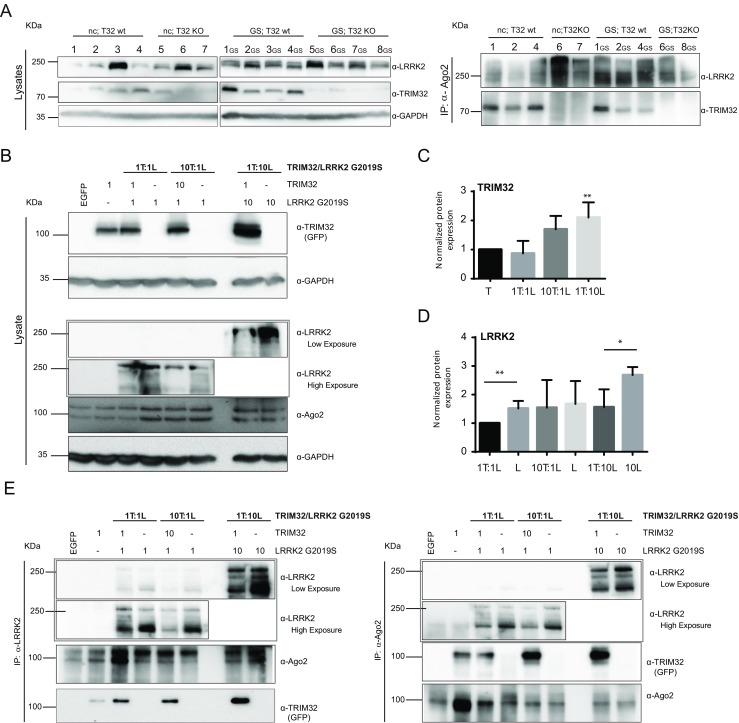
TRIM32 and LRRK2 do not antagonize for binding at the RISC. **a** On the *left panel*, brain lysates obtained from adult mice with the indicated genotypes are shown. The blots are probed with the indicated antibodies. Each *lane* correspond to an individual mice identified with unique numbers. Abbreviations: *nc* non-carrier, absence of mutations in the LRRK2 gene; *GS* LRRK2 G2019S mutation carriers; *T32wt* mice expressing TRIM32; *T32KO* mice lacking TRIM32 protein. On the *right panel*, immunoblots, probed with the indicated antibodies, after immunoprecipitation with an anti-Ago2 antibody, are shown. **b** HEK293T cells were transfected with different ratios of plasmids for the overexpression of the indicated constructs. Abbreviations: *T* GFP-TRIM32, *L* Flag-LRKK2 G2019S. Immunoblots from lysates of these cells showing the expression levels of LRRK2, TRIM32, and Ago2 are shown. In the case of LRRK2 detection, we show two different exposures of the same blot, due to the high difference in the expression levels when LRRK2 has been transfected in a 1:1 vs. 1:10 times ratio. **c** Densitometric quantification of TRIM32 levels normalized to GAPDH expression levels; statistical differences were determined compared with the control conditions in which TRIM32 has been transfected without the presence of LRRK2. **d** Densitometric quantification of LRRK2 levels normalized to GAPDH expression levels; statistical analysis was performed compared with the control conditions in which LRRK2-G2019S has been transfected without the presence of TRIM32. **e** Immunoblots from immunoprecipitations with anti-LRRK2 or anti-Ago2 antibodies from lysates of HEK293T cells, transfected as indicated in **b**, are shown. The blots were probed with the indicated antibodies. Uncropped blots with high exposure, probed with the anti-LRRK2 antibody, are shown in the supplementary information (Supplementary Fig. 2A)

In the next step, we wanted to assess whether the interaction of TRIM32 and LRRK2 with the Ago2-containing RISC is mutually dependent and whether the pathogenic LRRK2-G2019S mutation has any influence on this interaction. Therefore, we generated double-transgenic mice that either express the wild-type version of TRIM32 or present a TRIM32 knockout [23]. Additionally, these mice express either wild-type LRRK2 (non-carrier, nc) or LRRK2-G2019S [24]. We used brain lysates from these mice to conduct an IP with anti-Ago2 antibodies. However, regardless of the presence or absence of TRIM32, the Ago2-LRRK2 interaction was robustly present (Fig. 2a). Moreover, the interaction between Ago2 and TRIM32 was not influenced by the presence or absence of the LRRK2-G2019S mutation (Fig. 2a).

Both LRRK2 and TRIM32 can bind to Ago2 independently of each other but have reported opposite effects on the activity of the RISC. Due to that, we were aiming at elucidating whether they probably compete for binding to Ago2. In order to address this question, we expressed TRIM32 and LRRK2-G2019S in the ratio 1:1, 1:10, or 10:1 in HEK293T cells (Fig. 2b). Interestingly, we observed the highest levels of TRIM32 when it is co-expressed with a ten times excess of LRRK2 (Fig. 2b, c). We speculate that high LRRK2 levels might inhibit the degradation of TRIM32 via auto-ubiquitination and thereby increase its stability. As expected, highest LRRK2 expression levels were observed when it is expressed at a ten times excess (Fig. 2b, d). However, this is significantly reduced when it is co-expressed with TRIM32 (10× LRRK2, 1× TRIM32). This observation might indicate that TRIM32 downregulates LRRK2 when it is overexpressed, which probably is mediated via an ubiquitin ligase activity of TRIM32 towards LRRK2. However, we were not able to detect an effect of TRIM32 on the LRRK2 levels in the previous experiments (Fig. 1, Supplementary Fig. 1), indicating that this effect probably strongly depends on the levels in which both proteins are present. Strikingly, when immunoprecipitating LRRK2 or Ago2 from these cell lysates, we found that the interactions between Ago2 and TRIM32 as well as between Ago2 and LRRK2 are independent of their expression levels (Fig. 2e). Specificity of the immunoprecipitation assays was verified by control immunoprecipitations with IgG isotype-negative control antibodies (Supplementary Fig. 2B). Based on these results, we conclude that LRRK2 and TRIM32 do not exclude each other at the Ago2-binding position, indicating that they most likely bind to different domains of the Ago2 protein.

### TRIM32 Antagonizes the Inhibition of miRNA Activity Induced by Pathogenic Mutants of LRRK2

**Fig. 3:**
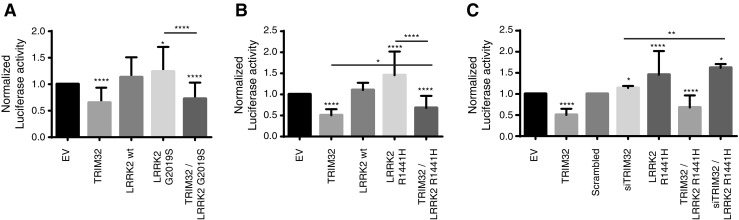
TRIM32 antagonizes the pathogenic LRRK2 mutant-induced inhibition of Let-7a activity. **a**–**c** N2a cells were transfected with dual-luciferase activity sensors for the activity of Let-7a. Additionally, the N2a cells were transfected with the indicated plasmids (*EV* empty vector). The diagrams show the detected normalized activity of the sensors. Statistical differences were determined with a Student *t* test

After showing that there is no binding antagonism between TRIM32 and LRRK2 (Fig. 2), we wanted to find out whether there might be a functional antagonism concerning miRNA activity regulation. Therefore, we co-expressed sensors for Let-7a activity together with TRIM32 and wild-type LRRK2 or the two pathogenic LRRK2 mutant forms G2019S and R1441H in N2a cells. As previously reported, we were able to see increased miRNA activity, represented by a reduced luciferase reporter activity, in the presence of TRIM32 (Fig. 3). While expression of wild-type LRRK2 has no significant effect on the activity of Let-7a, expression of either LRRK2-G2019S or LRRK2-R1441H significantly reduced the activity of Let-7a (represented by increased reporter activity, Fig. 3a, b). Strikingly, co-expression of TRIM32 together with LRRK2-G2019S or LRRK2-R1441H not only blocks their miRNA inhibition; it is even sufficient to induce activation of the miRNA Let-7a (Fig. 3a, b), although to a weaker extent than TRIM32 expression alone. Finally, we addressed the effect of a TRIM32 loss of function and observed that knockdown of TRIM32 inhibits the activity of Let-7a (Fig. 3c). Altogether, these data suggest that there is a functional antagonism between TRIM32 and pathogenic LRRK2 and that TRIM32 functions downstream of LRRK2.

### Pathogenic Mutant LRRK2 Inhibits TRIM32-Induced Neuronal Differentiation

**Fig. 4:**
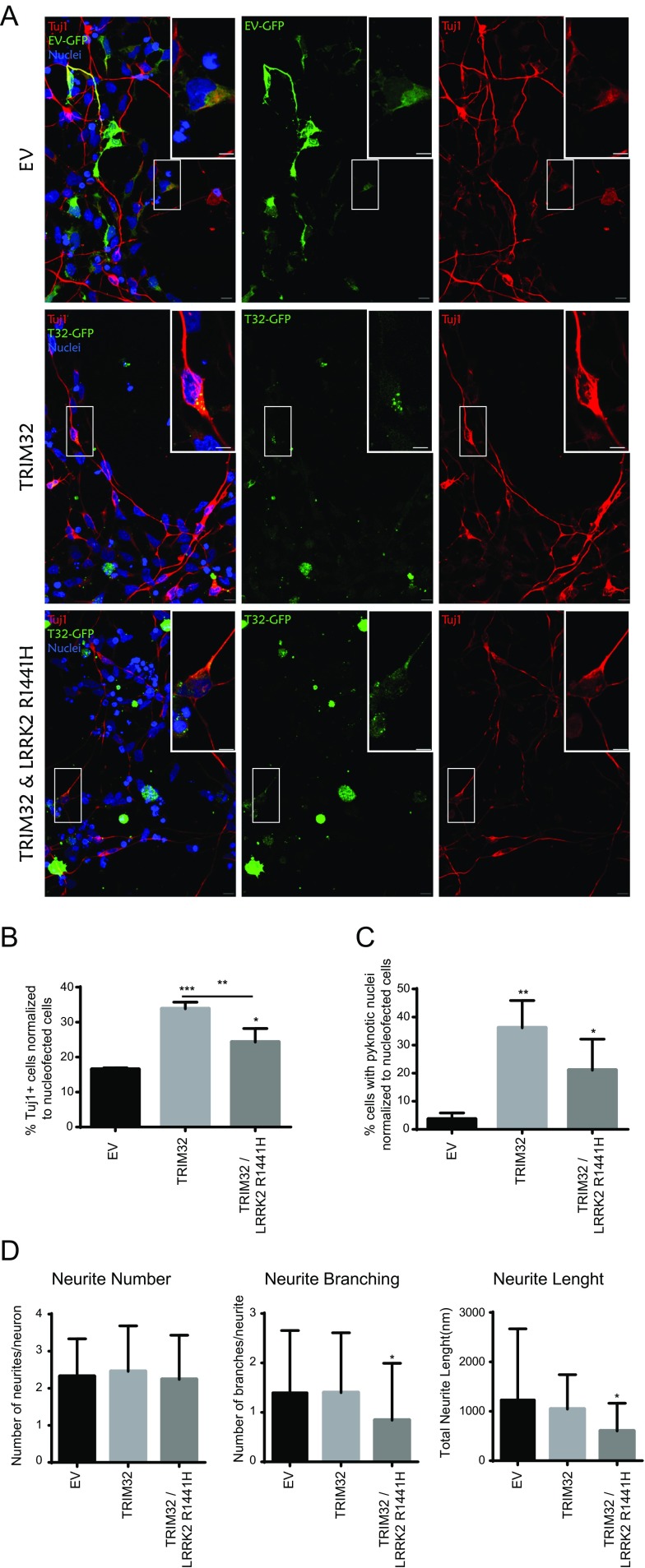
Pathogenic LRRK2 inhibits TRIM32-induced neuronal differentiation. **a**–**c** Neuroepithelial stem cells were nucleofected with plasmids for the expression of GFP, TRIM32-GFP, or TRIM32-GFP + Flag-LRRK2-R1441H. After fixation, nucleofected cells (*green*) were stained with antibodies against TuJ1 (*red*) indicative of neuronal differentiation. DNA was visualized by staining with Hoechst. Neuronal differentiation (**b**), pyknotic nuclei indicating cell death (**c**), and neurite complexity (neurite number, branching, and length) in transfected cells were quantified. Statistical differences were determined using an unpaired *t* test (*P < 0.05, **P < 0.01, ***P < 0.005)

TRIM32 is well known for its function during neuronal cell fate specification [12, 13, 18, 25]. Since we have demonstrated here that pathogenic LRRK2 has an antagonistic function to TRIM32 during miRNA activity regulation, we speculated whether this also influences TRIM32-induced neuronal differentiation. To address this question, we used a culture system of human neuroepithelial stem cells (NESCs) [26]. The NESCs were electroporated either with an empty vector only expressing GFP (EV, negative control), GFP-tagged TRIM32, or GFP-tagged TRIM32 together with Flag-tagged LRRK2-R1441H (Fig. 4). Electroporated cells were identified by GFP expression, and co-expression of LRRK2-R1441H was verified by staining with an anti-Flag antibody (Supplementary Fig. 3B). Neuronal differentiation was analyzed 48 h after electroporation by staining with the neuronal marker Tuj1. Additionally, we used Hoechst staining to analyze pyknotic nuclei, indicative of cell death. Previously, we have already shown that LRRK2 mutated at position 1441 inhibits neuronal differentiation [5]. As previously described, expression of TRIM32 was able to induce neuronal differentiation (Fig. 4a, b and supplementary Fig. 3A); in addition, expression of TRIM32 led to a marked increase in cell death (Fig. 4c and Supplementary Fig. 4). Both the induction of neuronal differentiation and the concomitant increase in cell death are significantly inhibited by co-expression of LRRK2-R1441H. Therefore, we conclude that pathogenic LRRK2 antagonizes not only the TRIM32-induced miRNA activity but also neuronal differentiation. Finally, since reduced neurite complexity is a well-known characteristic of in vitro cell culture models for PD [27], we also analyzed this feature. In the here used assay, expression of TRIM32 had no effect on neurite number, branching, or length. However, TRIM32 was also not able to antagonize the LRRK2-R1441H-induced reduction in neurite branching and length (Fig. 4d).

What do these results imply for the function of pathogenic LRRK2 during Parkinson’s disease? We are convinced that our here presented results support the hypothesis that PD has a strong neurodevelopmental component. Based on our results, we conclude that pathogenic LRRK2 inhibits the activity of miRNAs in neural progenitor cells during brain development and therefore interferes with the precise timing of neuronal differentiation. At a first glance, it is counterintuitive that pathogenic LRRK2 also reduces the level of cell death that is induced by TRIM32-mediated neuronal differentiation. However, cell death, particularly correctly timed, is an important aspect of brain development. Disturbing the correct dynamics of neuronal differentiation and cell death might lead to an altered neuronal network more prone to neuronal degeneration at later stages. Additionally, we here identify TRIM32 as an interesting new modifier for PD, which might be relevant as a novel target for future therapeutic approaches.

## Materials and Methods

### Cell Lines, Culture Conditions, and Transfections

Neuroblastoma (N2a) and human embryonic kidney (HEK) 293T cells were maintained in DMEM (Sigma) supplemented with 10% heat-inactivated fetal bovine serum (FBS), 200 mM l-glutamine, and penicillin/streptomycin in a 5% CO_2_-humidified atmosphere at 37 °C.

For protein analysis, cells were seeded onto non-coated plates at high density the day before transfection. The following day, cells were transfected with the indicated plasmids using TurboFect (Fermentas) according to the manufacturer’s recommendations.

In order to assess neuronal differentiation potential, we used human NESCs [26]. Cells were seeded onto matrigel-coated coverslips under maintenance culture conditions, and 18–24 h later, they were transfected in adherence conditions with a 4D-Nucleofector™ Y Unit (LONZA). Following the manufacturer’s instructions, cells were transfected with a total amount of 24 μg of DNA diluted into AD1 4D-Nucleofector™ Y solution using program ER-137. Combinations of pmaxGFP vector as control, pBABE-Flag, pcDNA3-GFP-Myc(N-Term)-TRIM32, and pCMV-Flag-LRRK2-R1441H were used for transfections. In order to induce neuronal differentiation, 24 h after transfection, culture media were completely exchanged with differentiation media containing 1 μl PMA, 10 μl hBDNF, 10 μl hGDNF, 100 μl ascorbic acid, 1 μl TGFb3, and 50 μl dbcAMP (10 ml). Cells were incubated for 60–72 h and then fixed in 4% PFA for 15 min at RT.

### Mice and Tissue Samples

Mouse husbandry was conducted in accordance with the existing Luxembourgish regulations for the protection of animals used for scientific purposes. TRIM32 +/− mice in a C57BL/6N background [23] were backcrossed with either LRRK2-G2019S or LRRK2 R1441G hemizygous mice (JAX® Mice) for at least five generations to generate the double-mutant animals with an enriched FvB background. Once a more pure FvB background was reached for all the genotypes used in this study we proceeded with the breedings to obtain the control and experimental animals. For that purpose, TRIM32 +/- mice, were crossed with TRIM32+/-; hemizygous LRRK2-G2019S or LRRK2-R1441G mice, obtaining hemizygous LRRK2-G2019S; TRIM32 -/- and hemizygous LRRK2-R1441G: TRIM32 -/- mice, respectively.

Genotyping of the animals was performed as previously described [23] or accordingly with the protocol defined by the provider (JAX).

Mouse brain samples were obtained from animals with the indicated genotypes and ages. For tissue collection, mice were euthanized according to the Luxembourgish Law for animal experimentation, and brains were dissected and deep frozen in liquid nitrogen and stored at −80 °C for subsequent protein extraction. For immunoblotting analysis, brain samples were homogenized using syringes of decreasing sizes in lysis buffer on ice until obtaining a cellular suspension that was subsequently treated as a cellular lysate.

### Western Blot and Immunoprecipitation

For Western blot and immunoprecipitation assays, protein extraction from brain lysates and cells was performed with lysis buffer consisting of 2% Triton X-100 and complete protease inhibitor cocktail (PIC) (Roche) in phosphate-buffered saline (PBS) supplemented with 3 μl/ml RNAse A. In order to detect protein-protein interactions, equal amounts of protein were incubated for 4 h with the indicated precipitating antibodies at 4 °C, followed by overnight incubation with protein-G agarose beads (GE Healthcare). Subsequently, protein complexes were eluted by boiling the samples for 5 min at 99 °C in protein sample buffer.

Protein lysates and protein complexes from the immunoprecipitation assays were resolved by SDS-PAGE, and immunoblotting was performed. Nitrocellulose membranes were incubated at 4 °C with the following primary antibodies: rabbit anti-LRRK2 (MJFF2(c41–2)) (Abcam, ab133474), rabbit anti-TRIM32 (#3149 and #3150, [17]), rabbit anti-GAPDH (D16H11) XP (Cell Signaling, #5174), and rabbit anti-Ago2/eIF2C2 (Abcam, ab32381). Membranes were incubated with the appropriate HRP-coupled secondary antibodies (GE Healthcare), and the enhanced chemiluminescence signal was detected with SuperSignal West Pico Chemiluminescent Substrate (Pierce, Thermo Scientific). Protein content was visualized with a STELLA 3200 Bio/Chemiluminescence modular imaging system (RAytest) device, acquiring several exposure times for each membrane.

Quantification was completed using ImageJ and its FIJI plugin; all data shown are normalized to the intensity of GAPDH, which had been used as loading control.

### Luciferase Assays

N2a cells were seeded at a density of 10^5^ cells per well in 24-well plates. The following day, cells were transfected using TurboFect (Fermentas) according to the manufacturer’s instructions. To determine the activity of miRNA Let-7a, each experimental condition contained 0.25 μg of pcDNA3.1D-Let-7a-1 [12] together with 0.2 μg of pmirGLO-let7 [28]. Up to 0.6 μg was completed with a 1:1 ratio of each of the indicated plasmids: pBABE-Flag, pcDNA3-GFP-Myc(N-Term)-TRIM32, pCMV-Flag-LRRK2-wt, pCMV-Flag-LRRK2-G2019S, pCMV-Flag-LRRK2-R1441H, pRNAT-H1.4/Retro-GFP(IRES)-scrambled sequence (siRNA12), pRNAT-H1.4/Retro-GFP(IRES)-TRIM32 (siRNA 2), and pRNAT-H1.4/Retro-GFP(IRES)-TRIM32 (siRNA 5) (GenScript). Forty-eight after transfection, cells were lysed and firefly and renilla luciferase activities were measured using the Dual-Luciferase Assay System (Promega) according to the manufacturer’s instructions in a Microplate Reader Infinite M200Pro-LCSB0414 (TECAN). The ratio of firefly luciferase (FL) activity to renilla luciferase (RL) was determined for each reaction, and all values were normalized to those of the empty vector.

### Immunostaining and Confocal Analysis

Fixed cells were permeabilized with 0.5% Triton X-100 in PBS, 15 min at RT, blocked with 10% FBS for 1 h at RT, and incubated o/n at 4 °C with antibodies against LRRK2 (Rabbit, Abcam, ab133474 (MJFF2(c41-2)), neuron-specific class III ß-tubulin (Tuj1) (mouse, Covance, PRB-435P). Immunofluorescent detection was carried out with the corresponding Alexa Fluor© 568 secondary antibodies, and nuclei were counterstained with Hoechst 33342 Solution (Invitrogen).

Tile scan pictures of random areas across the coverslips were acquired with a ×63 magnification in a confocal microscope LSM 710/Observer Z1-LCSB0451 (Zeiss). Quantification of the number of nuclei was done using ImageJ ITCN Plugin; however, the number of neurons and pyknotic nuclei was determined manually counting a minimum of 2000 cells for each experimental replicate and condition from three independent experiments. To determine statistical differences, unpaired two-tailed *t* test was performed.

### Statistical Analysis

In order to determine statistical differences in the different experiments, unpaired Student’s *t* test was performed. At least three independent experiments were analyzed in each case and *P* values smaller than 0.05 were considered significant.

## Electronic supplementary material

ESM 1 (DOCX 18 kb)

Supplementary Figure 1 TRIM32 interacts with LRRK2. A) HEK293T cells were transfected with plasmids for the overexpression of the indicated constructs. On the left panel immunoblots of the cell lysates, probed with the indicated antibodies, are shown. On the right panel immunoprecipitations with anti-Flag antibodies are shown. The blots are probed with the indicated antibodies. Abbreviations: CTRL: Control (untransfected cells). B) Densitometric quantification of LRRK2, Ago2 and TRIM32 levels normalized to GAPDH expression levels, corresponding to the blots from Fig. 1A is shown. (PDF 664 kb)

Supplementary Figure 2 TRIM32, LRRK2 and Ago2 form a complex. A) HEK293T cells were transfected with different ratios of plasmids for the overexpression of the indicated constructs. Abbreviations: T: GFP-TRIM32, L: Flag-LRRK2 G2019S. Immunoblots from lysates of these cells showing the expression levels of LRRK2 are shown. These blots represent the uncropped high exposure blots that are shown cropped in Fig. 2B and E. B) Lysates (L) obtained from adult mice expressing LRRK2 G2019S and wild type for TRIM32 were used for control immunoprecipitations with IgG isotype negative control antibodies (IP IgG). Two different mice (3GS and 4GS) are shown. The blots are probed with the indicated antibodies. (PDF 266 kb)

Supplementary Figure 3 Pathogenic LRRK2 inhibits TRIM32 induced neuronal differentiation. A) – B) Neuroepithelial stem cells were nucleofected with plasmids for the expression of GFP, TRIM32-GFP or TRIM32-GFP + Flag-LRRK2-R1441H. After fixation nucleofected cells (green) were stained with antibodies against TuJ1 (A, red) or Flag (B, red). DNA was visualized by staining with Hoechst. Low-magnification images are shown (A) and co-transfection is visualized (B). (PDF 36908 kb)

Supplementary Figure 4 Pathogenic LRRK2 also inhibits TRIM32 induced cell death. Neuroepithelial stem cells were nucleofected with plasmids for the expression of GFP, TRIM32-GFP or TRIM32-GFP + Flag-LRRK2-R1441H. After fixation nucleofected cells (green) were stained with Hoechst to visualize DNA. Pyknotic nuclei (box in the left panel, asterisk in the right panel) are shown. (PDF 1920 kb)
