# Supplementary material for: Parkinson’s Disease-Associated Mutant LRRK2-Mediated Inhibition of miRNA Activity is Antagonized by TRIM32
Source: Mol Neurobiol. 2017 May 15;55(4):3490–8. doi: 10.1007/s12035-017-0570-y (PMC5842508; doi:10.1007/s12035-017-0570-y)
Supplement: Supplementary file 1 — (DOCX 18 kb) [file 12035_2017_570_MOESM1_ESM.docx]

Parkinson’s disease associated mutant LRRK2 mediated inhibition of miRNA activity is antagonized by TRIM32.

- Supplementary information -

Laura Gonzalez-Cano, Ingeborg Menzl, Johan Tisserand, Sarah Nicklas, Jens C. Schwamborn

Luxembourg Centre for Systems Biomedicine (LCSB), University of Luxembourg

***Correspondence: Jens C. Schwamborn, Luxembourg Centre for Systems Biomedicine (LCSB), University of Luxembourg, 6, avenue du Swing, 4367 Belvaux, Luxembourg; E-mail: [jens.schwamborn@uni.lu](mailto:jens.schwamborn@uni.lu)

**Supplementary Figure Legends**

**Supplementary Figure 1: TRIM32 interacts with LRRK2.**

A) HEK293T cells were transfected with plasmids for the overexpression of the indicated constructs. On the left panel immunoblots of the cell lysates, probed with the indicated antibodies, are shown. On the right panel immunoprecipitations with anti-Flag antibodies are shown. The blots are probed with the indicated antibodies.

Abbreviations: CTRL: Control (untransfected cells)

B) Densitometric quantification of LRRK2, Ago2 and TRIM32 levels normalized to GAPDH expression levels, corresponding to the blots from Fig. 1A is shown.

**Supplementary Figure 2: TRIM32, LRRK2 and Ago2 form a complex.**

A) HEK293T cells were transfected with different ratios of plasmids for the overexpression of the indicated constructs. Abbreviations: T: GFP-TRIM32, L: Flag-LRRK2 G2019S. Immunoblots from lysates of these cells showing the expression levels of LRRK2 are shown. These blots represent the uncropped high exposure blots that are shown cropped in Fig. 2B and 2E.

B) Lysates (L) obtained from adult mice expressing LRRK2 G2019S and wild type for TRIM32 were used for control immunoprecipitations with IgG isotype negative control antibodies (IP IgG). Two different mice (3GS and 4GS) are shown. The blots are probed with the indicated antibodies.

**Supplementary Figure 3: Pathogenic LRRK2 inhibits TRIM32 induced neuronal differentiation.**

A) – B) Neuroepithelial stem cells were nucleofected with plasmids for the expression of GFP, TRIM32-GFP or TRIM32-GFP + Flag-LRRK2-R1441H. After fixation nucleofected cells (green) were stained with antibodies against TuJ1 (A, red) or Flag (B, red). DNA was visualized by staining with Hoechst. Low-magnification images are shown (A) and co-transfection is visualized (B).

**Supplementary Figure 4: Pathogenic LRRK2 also inhibits TRIM32 induced cell death.**

Neuroepithelial stem cells were nucleofected with plasmids for the expression of GFP, TRIM32-GFP or TRIM32-GFP + Flag-LRRK2-R1441H. After fixation nucleofected cells (green) were stained with Hoechst to visualize DNA. Pyknotic nuclei (box in the left panel, asterisk in the right panel) are shown.
