# Supplementary figures and images for: Parkinson’s Disease-Associated Mutant LRRK2-Mediated Inhibition of miRNA Activity is Antagonized by TRIM32
Source: Mol Neurobiol. 2017 May 15;55(4):3490–8. doi: 10.1007/s12035-017-0570-y (PMC5842508; doi:10.1007/s12035-017-0570-y)

A

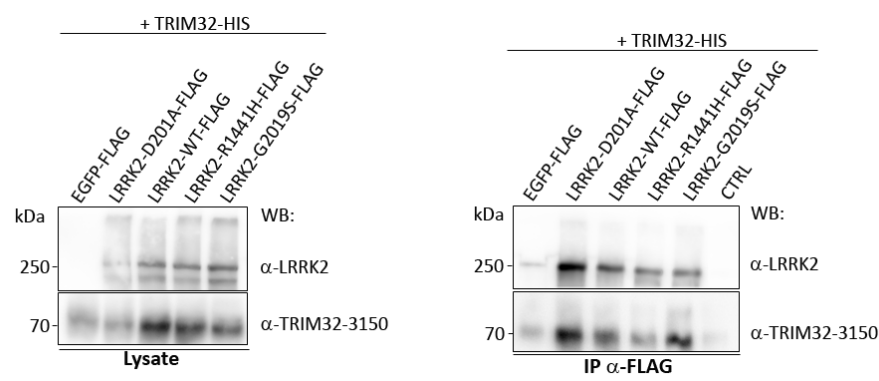

B

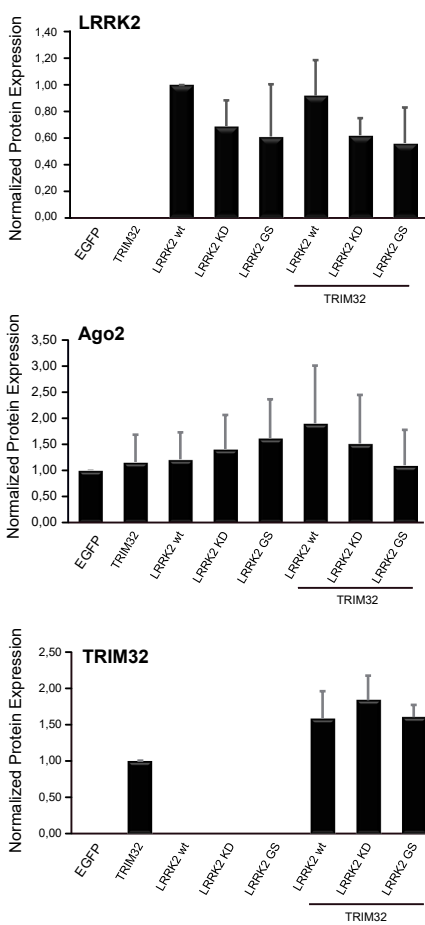

Gonzalez-Cano et al, Supplementary Figure 1

Supplement: Supplementary file 2 — TRIM32 interacts with LRRK2. A) HEK293T cells were transfected with plasmids for the overexpression of the indicated constructs. On the left panel immunoblots of the cell lysates, probed with the indicated antibodies, are shown. On the right panel immunoprecipitations with anti-Flag antibodies are shown. The blots are probed with the indicated antibodies. Abbreviations: CTRL: Control (untransfected cells). B) Densitometric quantification of LRRK2, Ago2 and TRIM32 levels normalized to GAPDH expression levels, corresponding to the blots from Fig. 1A is shown. (PDF 664 kb) [file 12035_2017_570_MOESM2_ESM.pdf]

A

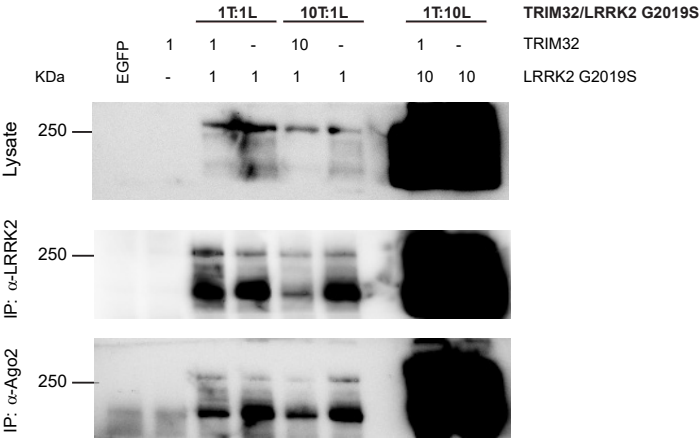

B

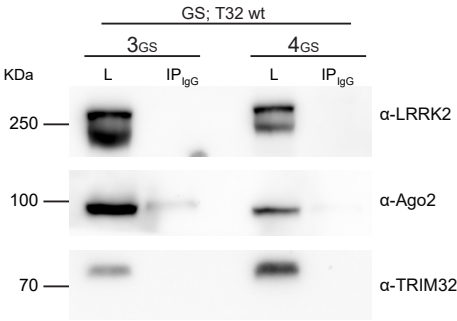

Supplement: Supplementary file 3 — TRIM32, LRRK2 and Ago2 form a complex. A) HEK293T cells were transfected with different ratios of plasmids for the overexpression of the indicated constructs. Abbreviations: T: GFP-TRIM32, L: Flag-LRRK2 G2019S. Immunoblots from lysates of these cells showing the expression levels of LRRK2 are shown. These blots represent the uncropped high exposure blots that are shown cropped in Fig. 2B and E. B) Lysates (L) obtained from adult mice expressing LRRK2 G2019S and wild type for TRIM32 were used for control immunoprecipitations with IgG isotype negative control antibodies (IP IgG). Two different mice (3GS and 4GS) are shown. The blots are probed with the indicated antibodies. (PDF 266 kb) [file 12035_2017_570_MOESM3_ESM.pdf]

A

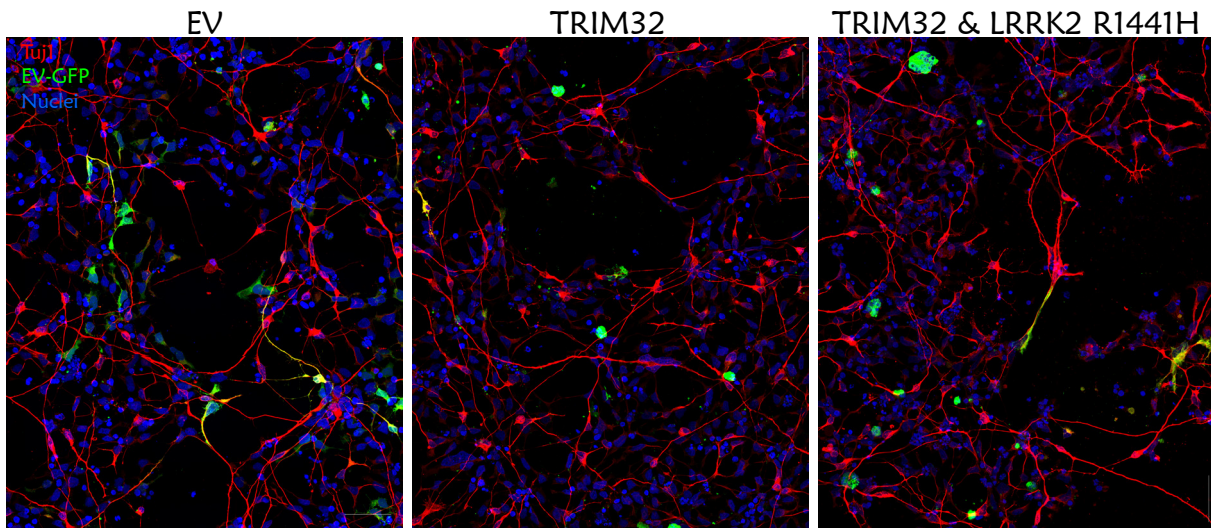

B

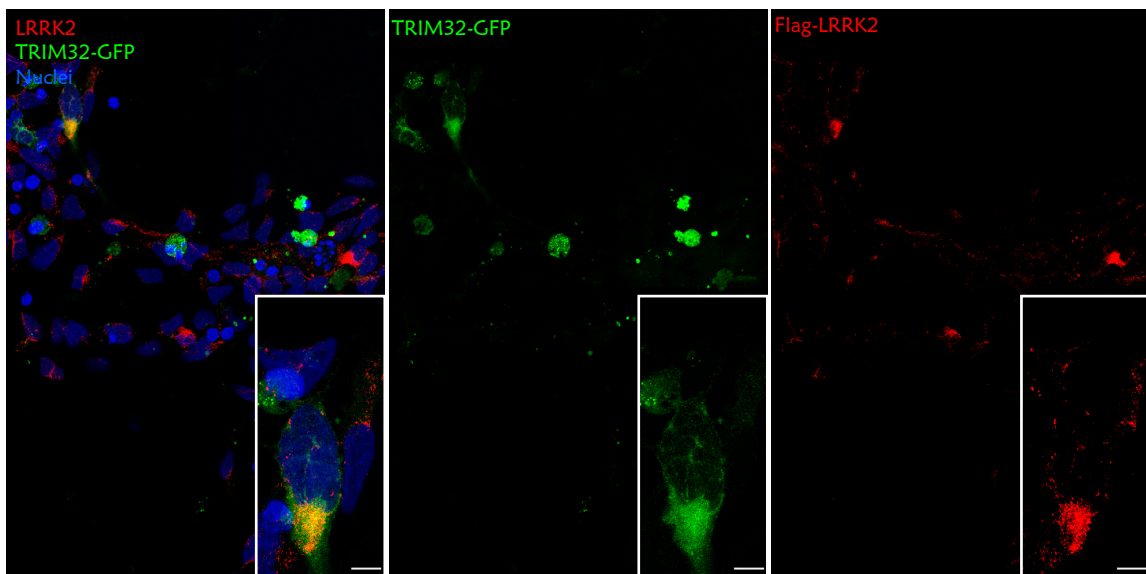

Supplement: Supplementary file 4 — Pathogenic LRRK2 inhibits TRIM32 induced neuronal differentiation. A) – B) Neuroepithelial stem cells were nucleofected with plasmids for the expression of GFP, TRIM32-GFP or TRIM32-GFP + Flag-LRRK2-R1441H. After fixation nucleofected cells (green) were stained with antibodies against TuJ1 (A, red) or Flag (B, red). DNA was visualized by staining with Hoechst. Low-magnification images are shown (A) and co-transfection is visualized (B). (PDF 36908 kb) [file 12035_2017_570_MOESM4_ESM.pdf]

A

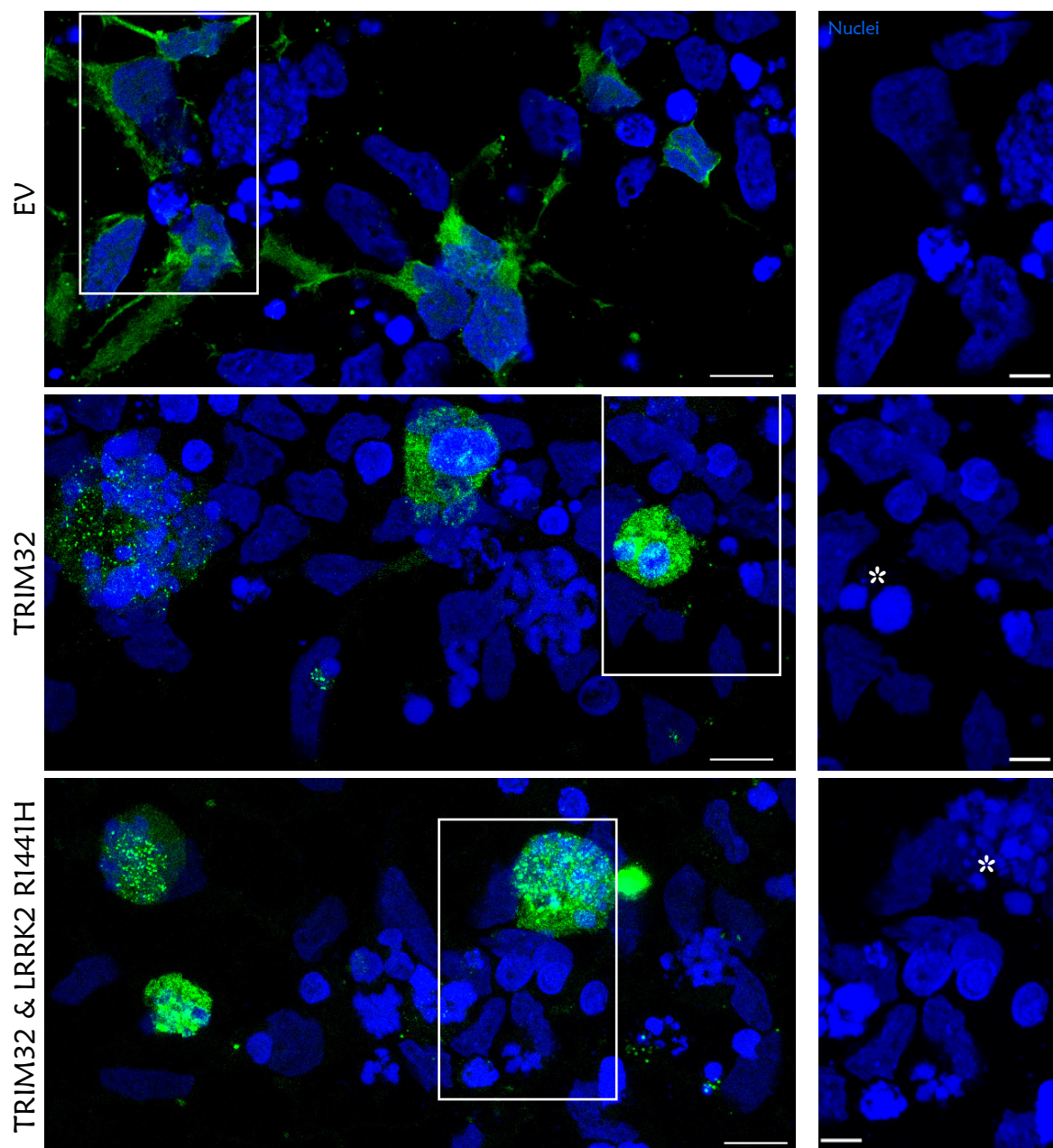

Gonzalez-Cano et al, Supplementary Figure 4

Supplement: Supplementary file 5 — Pathogenic LRRK2 also inhibits TRIM32 induced cell death. Neuroepithelial stem cells were nucleofected with plasmids for the expression of GFP, TRIM32-GFP or TRIM32-GFP + Flag-LRRK2-R1441H. After fixation nucleofected cells (green) were stained with Hoechst to visualize DNA. Pyknotic nuclei (box in the left panel, asterisk in the right panel) are shown. (PDF 1920 kb) [file 12035_2017_570_MOESM5_ESM.pdf]
